# TNF-α-mediated podocyte injury via the apoptotic death receptor pathway in a mouse model of IgA nephropathy

**DOI:** 10.1080/0886022X.2022.2079527

**Published:** 2022-07-14

**Authors:** Qiang Wan, Jiabao Zhou, Yansheng Wu, Liqiang Shi, Weiwei Liu, Jiaoying Ou, Jiandong Gao

**Affiliations:** a Department of Nephrology, Shuguang Hospital Affiliated to Shanghai University of Traditional Chinese Medicine; TCM institute of kidney disease, Shanghai University of Traditional Chinese Medicine, Key Laboratory of Liver and Kidney Diseases (Shanghai University of Traditional Chinese Medicine), Ministry of Education; Shanghai Key Laboratory of Traditional Chinese Clinical Medicine, Shanghai, China; b Department of Nephrology, Beilun Traditional Chinese Medicine Hospital, Ningbo, China; c Preventive treatment of disease center, Shuguang Hospital Affiliated to Shanghai University of Traditional Chinese Medicine, Shanghai, China

**Keywords:** IgA nephropathy, TNF-α, podocyte, apoptosis, death receptor pathway

## Abstract

**Background:**

IgA nephropathy (IgAN) is the most common primary glomerular disease worldwide and it is characterized by mesangial IgA deposits. Proteinuria is a common clinical feature of IgAN, which has a critical connection to podocyte injury and has been used as a clinical prognostic factor for IgAN. Evidence has shown that TNF-α released from mesangial cells may lead to podocyte apoptosis.

**Methods:**

Forty male BALB/c mouse were randomly divided into the control group and IgAN group. A mice model of IgAN was developed by oral administration of bovine serum albumin (BSA) combined with Staphylococcus Enterotoxin B (SEB) tail vein injection. Urinary protein concentrations, renal function, renal morphological, IgA deposition, apoptosis situation, and the mRNA and protein expression of nephrin, podocin, TNF-α, TNFR1, caspase-8 and caspase-3, were detected after 12 weeks.

**Results:**

BSA and SEB can successfully establish an IgAN mouse model, and the main pathological changes are the IgA immune complex deposition in the mesangial area. The gene and protein expression levels of nephrin and podocin were found to be downregulated, and death receptor pathway-related indicators were upregulated, and they were involved in TNF-α-activated podocyte injury and apoptosis in IgAN mice.

**Conclusion:**

TNF-α may play an important role in the pathogenesis of podocyte apoptosis in IgAN, and its effects may be mediated through the apoptotic death receptor pathway.

## Introduction

IgA nephropathy (IgAN) has become the most common primary glomerular disease worldwide since it was first reported by Berger in 1968 [1]. Among patients who have been definitively diagnosed with IgAN by renal biopsy, 10–30% progress to end-stage renal disease within 10 years after diagnosis [2]. The clinical manifestations include a wide range of symptoms and signs, from hematuria or proteinuria to severe hypertension due to renal failure. Mesangial polymeric IgA deposition is the main pathogenetic mechanism of IgAN [3], which triggers the proliferation of mesangial cells, and increases the synthesis of extracellular matrix and immune cell infiltration [4]. The commonly used IgAN animal models mainly include spontaneous, secondary and immune induction. Although rodent IgA differs from human IgA1, a variety of murine models have been developed, which may contribute to the study of the complex pathogenesis of IgA nephropathy from different perspectives [5].

Podocytes are a highly specialized type of cell positioned at the outer surface of capillaries and in the vicinity of Bowman's capsule. The filtration barrier is made up of glomerular epithelium, the glomerular basement membrane (GBM) and the slit diaphragm formed by the foot processes of podocytes [6]. These play an important role in preventing plasma proteins from entering the urine [7–9]. Podocyte injury is a factor underlying the development of proteinuria and glomerulopathies and has been used as a clinical prognostic factor for IgAN [10,11]. There is no evidence of any known IgA receptors in podocytes, and pathogenetic IgA is only deposited in the mesangial area of glomeruli [7–10]. Therefore, the factor responsible for protein leakage from the blood vessels remains elusive. Wang *et al* found that serum IgA1 from patients with IgAN may induce podocyte apoptosis through direct and indirect pathways [12].

Apoptosis, a genetically controlled programmed cell death pathway, plays a key role in tissue development, homeostasis and disease. Different death stimuli can cause apoptosis, which can be mediated through either intrinsic or extrinsic pathways [13]. The extrinsic apoptotic pathway, also known as the death receptor pathway, is initiated by ligands binding to death receptors embedded in the cell membrane. TNF receptor1(TNFR1) functions as the receptor of death ligand TNF; when TNF-α binds with TNFR1 to form ligand-receptor complex recruits and activates death-inducing signaling complex (DISC), which activates initiator caspase (caspase-8), it then cleaves and activates the executioner caspase (caspase-3), ultimately leading to apoptosis [14].

One *in vitro* study suggested that mesangial cell-derived TNF-α upregulated TNFR1 and TNFR2 expression in podocytes in patients with IgAN, and revealed that TNF-α causes apoptosis through binding with TNFR1 [7,15,16]. However, the mechanism and related pathway through which TNF-α causes podocyte apoptosis remains unknown. Therefore, it was hypothesized that TNF-α released from mesangial cells after IgA1 deposition, which serves as the ligand of the death pathway mediating cell apoptosis may play a role in regulating podocyte apoptosis in IgAN through the TNF/TNFR1-/caspase-8/caspase-3 death pathway. The aim of the present was to investigate the role of apoptosis in glomerular podocytes using *in vivo* models.

## Materials and methods

### Experimental animals

The present study has been approved by the institutional ethics committee of Shanghai University of Traditional Chinese Medicine (SHUTCM) for studies in mice (animal certification no.: 2008001662170; animal ethics registration no.: SZY201607002) and all experiments were conducted in accordance with the ethical standards, the Declaration of Helsinki and the related national and international guidelines [17]. A total of 40 SPF grade male BALB/c mice, aged 6–8 weeks and weighing 18–20 g, were purchased from the Shanghai Laboratory Animal Center, Co., Ltd. and housed at the SHUTCM experimental animal center. The mice were randomly divided into two groups (*n* = 20 per group).

### Model preparation

In this experiment, the heterologous protein bovine serum albumin, low endotoxin (BSA; cat. no. 9048-46-8; Yeasen Science and Technology Co., Ltd.)gavage, and the Staphylococcus Enterotoxin B (SEB; Academy of Military Medical Sciences, Beijing, China) tail vein injection method were used to induce the IgAN mouse animal model [18]. After adaptive feeding for 1 week, the mice in the control group were given normal saline (0.5 mL every day), and the model group mice were given 0.1% BSA acidified water (0.5 mL every other day) since the beginning of the experiment. The mice were injected with 2% BSA buffer (0.2 mL) at the beginning of the 6th week of the experiment, once per day for 3 consecutive days. BSA gavage was stopped at the 9th week of the experiment, and a tail intravenous injection of 0.1 mg/ml SEB buffer (0.2 mL) was initiated once per week for 3 weeks. The control group mice were given a tail intravenous injection of an equal volume of saline. The total duration of the experiment was 12 weeks.

### Sample collection and laboratory measurements

The body weight of the mice was recorded every 3 weeks. At the end of the experiment, 24-h urine samples were collected. Then the mice were sacrificed *via* cervical dislocation to collect blood samples and the bilateral kidneys. The blood and urine samples were submitted to Shuguang Hospital for accurate measurement of serum urea, serum creatinine (SCr), uric acid (UA) and urinary protein concentrations using the Beckman Coulter AU5800 automated analyzer (Beckman Coulter, Inc.). The 24-h urine protein was calculated by urinary protein concentration x urinary volume [19].

### Immunofluorescence

The kidneys were embedded in the Tissue-Tek O.C.T compound (Sakura Finetek USA, Inc.), frozen and cut on Leica CM3050S cryostat microtome (Leica Microsystems GmbH) into 6-µm sections. For IgA immunofluorescence, the sections were incubated with goat anti-mouse IgA Secondary Antibody (FITC) (1:100; cat. no. NB7503; Novus Biologicals, LLC).

Double immunofluorescence staining for nephrin (1:250; cat. no. BAF3159; R&D Systems, Inc.) and cleaved caspase-3 (1:400; cat. no. 9661; Cell Signaling Technology, Inc.) was carried out to examine the location of apoptosis in kidney glomerular paraffin sections. The sections were deparaffinized in xylene (2 × 5 min), hydrated with 100% ethanol (2 × 3 min) and hydrated with 95% ethanol (1 min). The membrane was incubated with 1% BSA in PBST (0.1% Tween-20) for 30 min to block the unspecific binding of the antibodies, in the mixture of two primary antibodies (diluted at 1:100) in 1% BSA in PBST in a humidified chamber for overnight at 4 °C, and with the mixture of two secondary antibodies, Alexa Fluor conjugated mouse anti-goat IgG (1:1000; cat. no. bs-0294W; Beijing Biosynthesis Biotechnology Co., Ltd.) and FITC conjugated goat anti-mouse IgG (1:1000; cat. no. bs-0296G; Beijing Biosynthesis Biotechnology Co., Ltd.)in 1% BSA for 1 h at room temperature in the dark.

### Renal morphometrics

The kidneys were fixed in neutral buffered formalin (10%), embedded in paraffin, cut into 3-µm, and stained with H&E (cat. no. G1005; Servicebio Science and Technology Co., Ltd.) for 5 min, periodic acid Schiff (cat. no. G1008; Servicebio Science and Technology Co., Ltd.) for 30 min and Masson’s trichrome stain (cat. no. G1006; Servicebio Science and Technology Co., Ltd.) for 10 min at room temperature. To observe renal ultrastructure, kidney tissues were fixed in 3% glutaraldehyde, postfixed in 1% osmium tetroxide, imbued with uranyl acetate, and embedded in epoxy resin. The specimen was cut into thin sections (50–60 nm) and examined under a transmission electron microscope (Tecnai G2 Bio TWIN; FEI Ltd.).

### TUNEL assay

*In Situ* Cell Death Detection kit (peroxidase and fluorescein; Roche Diagnostics) was used to detect apoptosis according to the manufacturer’s protocol. The kidneys were fixed with 10% neutral buffered formalin for 24 h at room temperature, paraffin-embedded, cut into 3-µm sections. After dewaxing with xylene, sections were incubated with proteinase K for 15 min at room temperature. Then the sections were incubated with dUTP and TdT in a humidified chamber for 60 min at room temperature and subsequently treated with converter peroxidase for 30 min and rinsed in PBS. After that, the sections were counterstained with DAPI for nuclear labeling for 5 min at room temperature and observed by fluorescence microscope. In addition, the sections were treated with DAB for 10 min and hematoxylin staining was counterstained for 2 min at room temperature. Then the sections were dehydrated with a graded series of alcohol and sealed with neutral balsam and observed by a light microscope. The data were collected using Image-Pro Plus 6.0 software (Media Cybernetics, Inc.) and the rate of cell apoptosis in glomeruli was calculated.

### Western blotting

The kidney cortex was homogenized in RIPA lysis buffer (cat. no. P0013B; Beyotime Institute of Biotechnology), and the protein concentrations were measured *via* a Pierce BCA Protein Assay kit (cat. no. 23225; Thermo Fisher Scientific, Inc.). Equal amounts of total protein (30 µg) were separated onto 612% SDS-PAGE and electrophoretically transferred to polyvinylidene difluoride membranes (Millipore, Sigma). The membranes were blocked with 5% skimmed milk for 2 h, and incubated with specific primary antibodies overnight at 4 °C: Podocin (1:500; cat. no. P0372; Sigma-Aldrich; Merck KGaA), nephrin (1:1,000; cat. no. BAF3159; R&D Systems, Inc.), GAPDH (1:1,000; cat. no. 2118; Cell Signaling Technology, Inc.), TNF-α (1:2,000; cat. no. ab9739; Abcam), caspase-8 (1:1,000; cat. no. ab2590; Abcam), caspase-3 (1:1,000; cat. no. ab184787; Abcam) and TNFR1 (1:1,000; cat. no. sc-8436; Santa Cruz Biotechnology, Inc.). Immunoreactive bands were detected using horseradish peroxidase (HRP)-conjugated goat anti-rabbit (1:5,000; cat. no. 111-035-003; Jackson ImmunoResearch Laboratory, Inc.) or goat anti-mouse IgG (1:5,000; cat. no. 115-035-003; Jackson ImmunoResearch Laboratory, Inc.) as the secondary antibody. After further washing, the proteins in the membrane were detected using Beyo ECL Plus (cat. no. P00185; Beyotime Institute of Biotechnology). Immunoreactive bands were visualized using a photodope-HRP western blotting detection system (cat. no. 7003; Cell Signaling Technologies, Inc.) and quantified through densitometry by using Quantity One software (Bio-Rad Laboratories, Inc.).

### Reverse transcription-quantitative PCR (RT-qPCR)

Total RNA was extracted from the kidneys of mice using TRIzol® reagent (Takara Bio, Inc.) and cDNA was synthesized in 20-µl reactions using the Superscript II reverse transcriptase kit (Takara Bio, Inc.). The expression levels of mRNAs were evaluated by qPCR with SYBR-Green reaction kit (cat. no. a25742; Applied Biosystems; Thermo Fisher Scientific, Inc.) and the ABI Step One Plus system (Applied Biosystems; Thermo Fisher Scientific, Inc.) and normalized against the expression of the GAPDH gene The amplification conditions were as follows: 50 °C for 2 min and 95 °C for 2 min followed by 40 cycles of 95 °C for 15 s, 60 °C for 1 min. After that, a melting curve analysis was performed. The primers used for the RT-PCR were designed and synthesized by Sangon Biotechnology Co., Ltd., and they are listed in Table 1. The data obtained were analyzed using the 2^-ΔΔCq^ method [20].

**Table 1.** PCR primer sequence.

Gene name		Primer sequence (5’ to 3’)	Product length
Podocin	FORWARD	CCGCTGCATTGAGAATGGAC	485
	REVERSE	GACTGGGGATGGTTCAGGAC	
Nephrin	FORWARD	CGCACGGTAGAGGATGTCTC	267
	REVERSE	TATGCAGGGTAGCTCCAGGT	
TNF-α	FORWARD	TCTTCAAGGGACAAGGCTGC	587
	REVERSE	CCTGACCACTCTCCCTTTGC	
TNFR1	FORWARD	GGCTCTGCTGATGGGGATAC	361
	REVERSE	CAGGTAGCGTTGGAACTGGT	
Caspase-8	FORWARD	GAGCCTCAAAATGGCGGAAC	441
	REVERSE	AGGGAAGGGCACTTTGAACC	
Caspase-3	FORWARD	CTCTGGTACGGATGTGGACG	185
	REVERSE	CCCCTTCATCACCATGGCTT	
GAPDH	FORWARD	GGTTGTCTCCTGCGACTTCA	300
	REVERSE	CCCTAGGCCCCTCCTGTTAT	

### Statistical analysis

All data are expressed as means ± SD. Data normality was verified by the Shapiro-Wilk test. The differences between the two sets of values were analyzed using Student’s t-test. *P*-values quoted were two-tailed and *p* < 0.05 was considered to indicate a statistically significant difference. The figures were prepared using GraphPad Prism 5.0 (GraphPad Software, Inc.).

## Results

### Characteristics of IgAN mice

**Figure 1.**
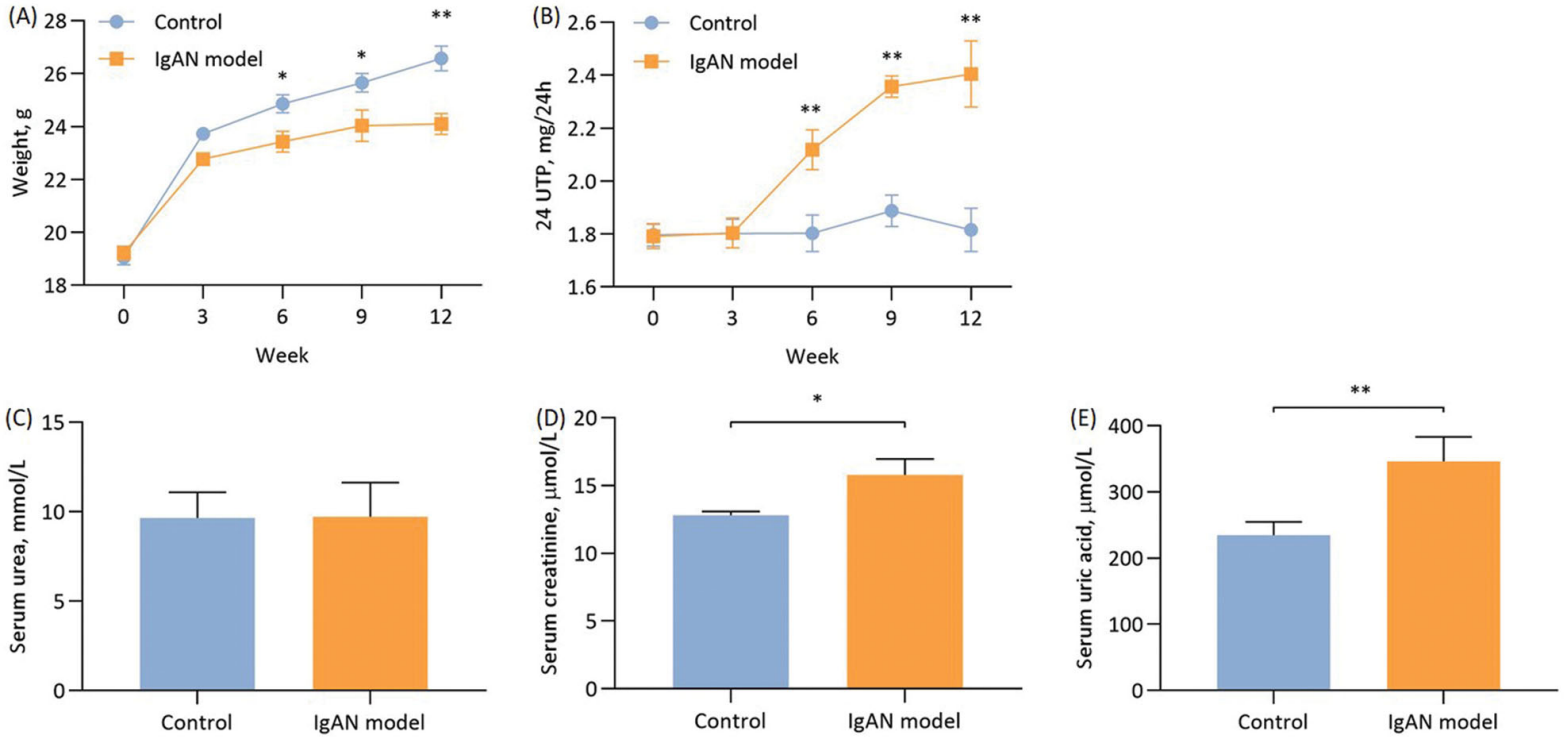
Characteristics of IgAN mice. (A) Changes in mouse weight and (B) 24-h urine protein over 12 weeks (*n* = 6). (C) Serum urea, serum creatinine (D) and uric acid levels (E) at the end of the 12 weeks (*n* = 6). Data are expressed as mean ± SD. *p < 0.05, **p < 0.01 vs. control group. IgAN, IgA nephropathy.

After the 12-week experimental period, the mice in the model group exhibited varying degrees of apathy, aversion to cold, loss of appetite and weight loss compared with the control group. Compared with the control group, there was a significant difference in weight (<0.05; Figure 1a) and 24-h urinary protein (*p* < 0.01; Figure 1b) at the 6th week of the experiment *p*. Furthermore, there was a significant difference in the weight and 24-h urinary protein at the end of the experiment in the model group compared with the control group (*p* < 0.01; Figure 1a, b).

Control group mice had mean serum urea of 9.65 ± 1.43 mmol/l, which was not significantly different from the mean serum urea value of 9.72 ± 1.90 mmol/l in the IgAN mice (*p* > 0.05; Figure 1c). The SCr in the control group mice was 15.78 ± 4.21 µmol/l, which was statistically significantly different from the control group SCr of 12.81 ± 1.24 µmol/l (*p* < 0.01; Figure 1d). In addition, the serum UA levels exhibited statistically significant differences between the control and model group mice (*p* < 0.01; Figure 1e).

**Figure 2.**
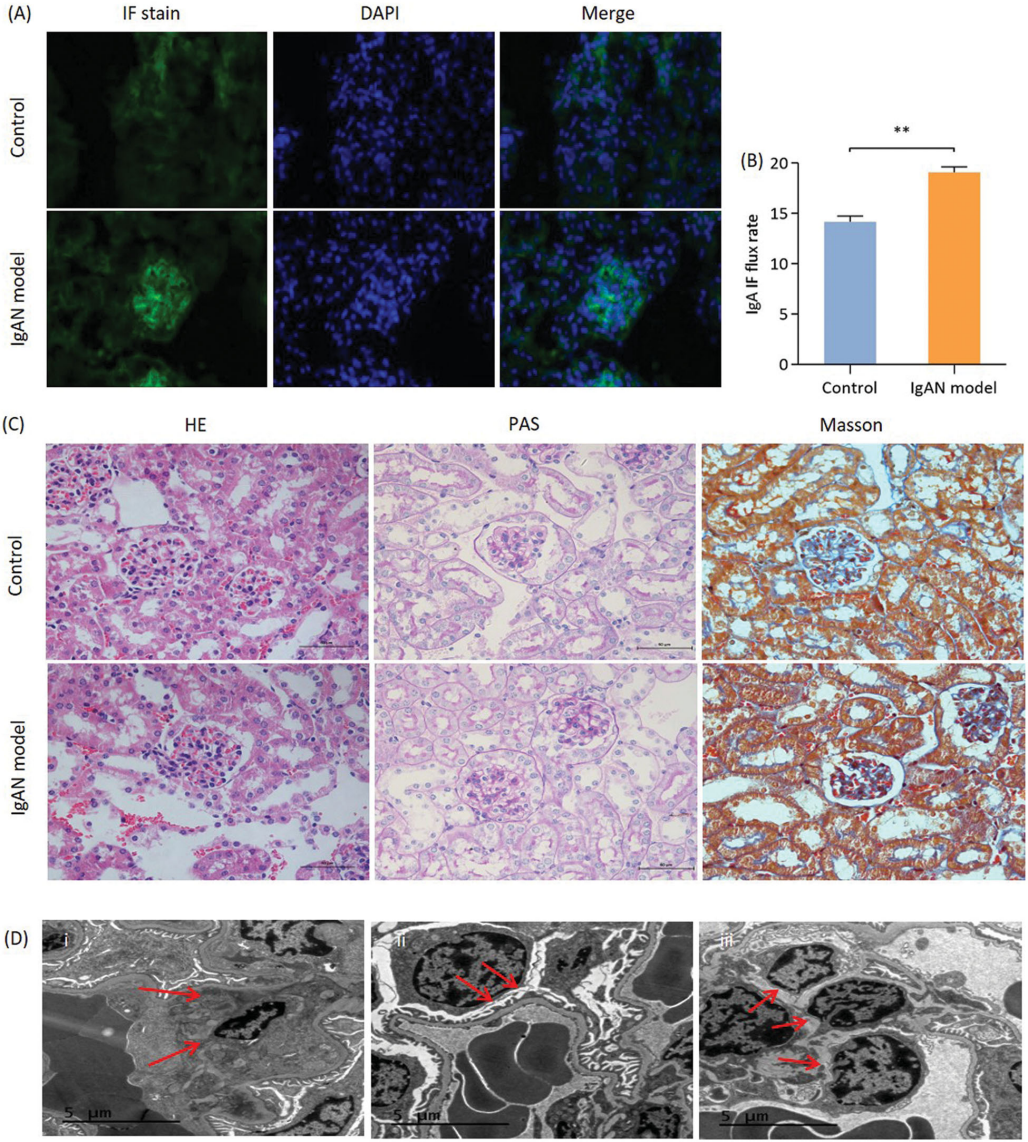
Renal pathological changes in IgAN mice. (A) Representative images of immunofluorescence staining (magnification, x 400). (B) Quantitative analysis of IgAN flux rate. Data are expressed as mean ± SD. (C) Representative histological images of sections stained (with H&E, PAS and Masson’s trichrome) stain (magnification, x400). (D) Electron microscopic images of IgAN mouse kidney sections. (i) Imaging of the mesangial area in the glomerulus. Red arrows, electron-dense granules. (ii) Imaging of the GBM and podocytes. Red arrows, podocyte apoptosis accompanied with foot process fusion. (iii) Imaging of mesangial area. Red arrows, the proliferation of mesangial cells and mesangial matrix. Scale bars, 5 µm. *p < 0.05, **p < 0.01 vs.control group. IgAN, IgA nephropathy; GBM, glomerular basement membrane; PAS, periodic acid-Schiff.

IgA immunofluorescence intensity was used to detect the expression of IgA deposition in renal glomeruli. The results demonstrated that the immunofluorescence intensity of IgA deposits observed in the model group was significantly higher compared with that in the control group (*p* < 0.01; Figure 2a, b).

An increased number of cell nuclei in the glomeruli was shown by performing H&E staining and the proliferation of mesangial cell and mesangial matrix were shown on PAS-stained sections. Mesangial expansion with increased extracellular matrix to different degrees, partly with glomerular capsule adhesion and partly focal hyperplasia or focal segmental sclerosis changes, were observed in severe cases in the glomeruli of model group mice. The renal tubulointerstitial lesions were consistent with mild to moderate mesangial changes, whereas there were no obvious abnormalities in the glomeruli of the control group mice. Some cases of advanced glomerulosclerosis in IgAN model group mice were paralleled by extensive tubular atrophy and interstitial fibrosis (Figure 2c). Electron microscopic examination revealed IgA-containing immune complexes deposited in the mesangial area, with mesangial cell and matrix proliferation and podocyte apoptosis in the IgAN model group mice (Figure 2d).

### Podocyte expression of podocin and nephrin in IgAN mice

**Figure 3.**
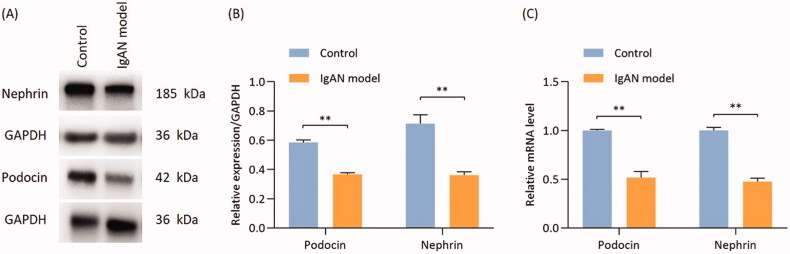
Proteins and mRNA expressions of podocin and nephrin are significantly reduced in IgAN mouse kidneys. (A) Western blotting results and (B) relative protein expression of nephrin and podocin. (C) Transcription levels of nephrin and podocin (*n* = 3). Data are expressed as mean ± SD. *p < 0.05, **p < 0.01 vs. control group. IgAN, IgA nephropathy.

The expression of the podocyte marker proteins podocin and nephrin was quantified by Western blotting and RT-qPCR. The protein and gene expressions of podocin and nephrin were found to be significantly reduced in IgAN mice as compared with those in the control group (*p* < 0.01; Figure 3a–c).

### Podocyte apoptosis in IgAN mice

**Figure 4.**
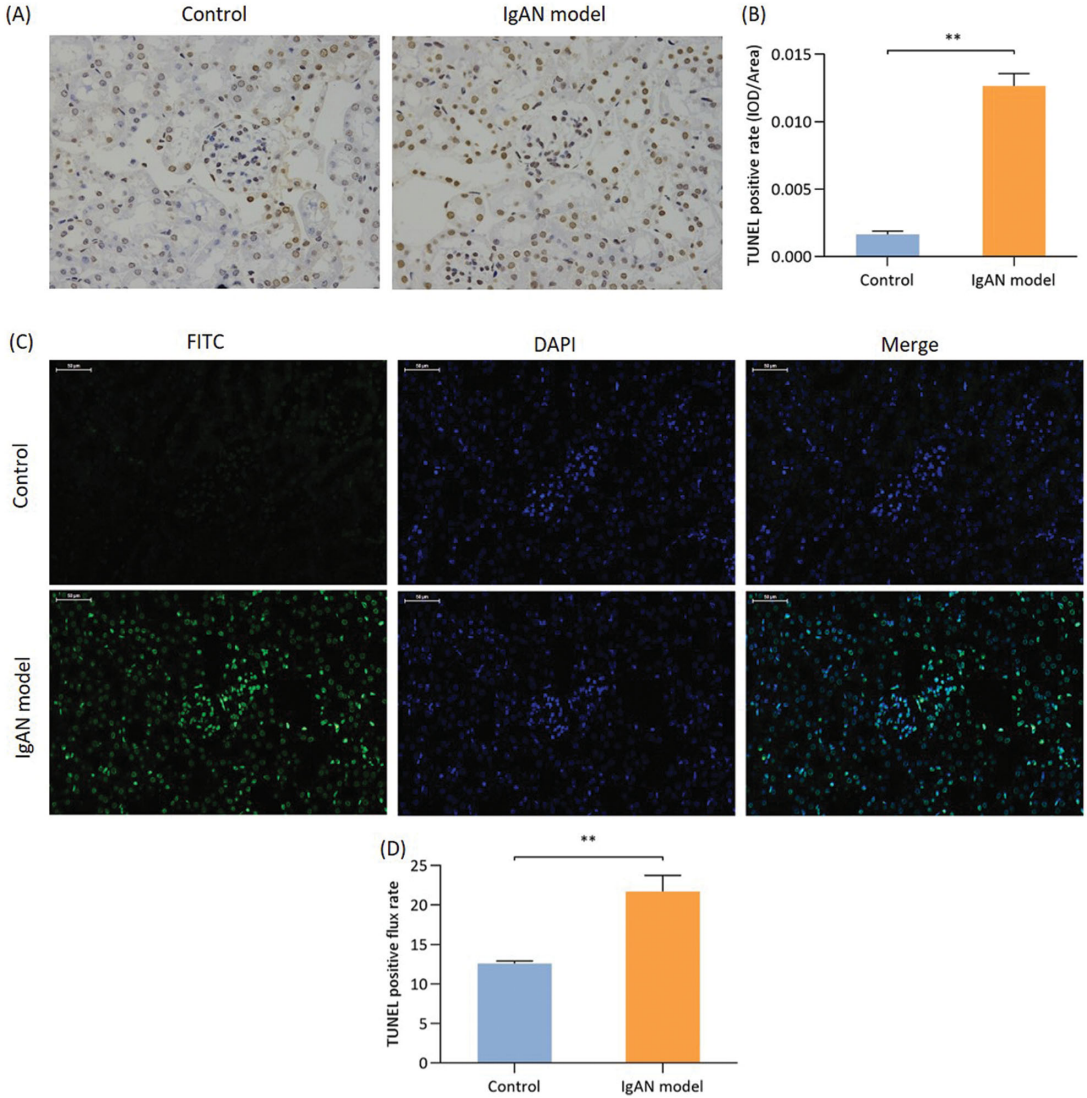
Podocyte apoptosis in IgAN mice. (A) TUNEL (biotin-labeled peroxidase) detection (magnification, x400) and the (B) results were quantified. (C) TUNEL method (FITC labeling) method (magnification, x400) and (D) quantification of the results. Data are expressed as mean ± SD. vs. control group. IgAN, IgA nephropathy.

To further determine the association between podocyte injury and apoptosis, the TUNEL assay was used, which was labeled by DAB and FITC through DNA fragmentation. The number of TUNEL-stained apoptotic cells labeled with either DAB or FITC was higher in the IgAN group compared with that in the control group (*p* < 0.01; Figure 4a–d).

### Co-localization of nephrin and caspase-3 in podocyte apoptosis in IgAN mice

**Figure 5.**
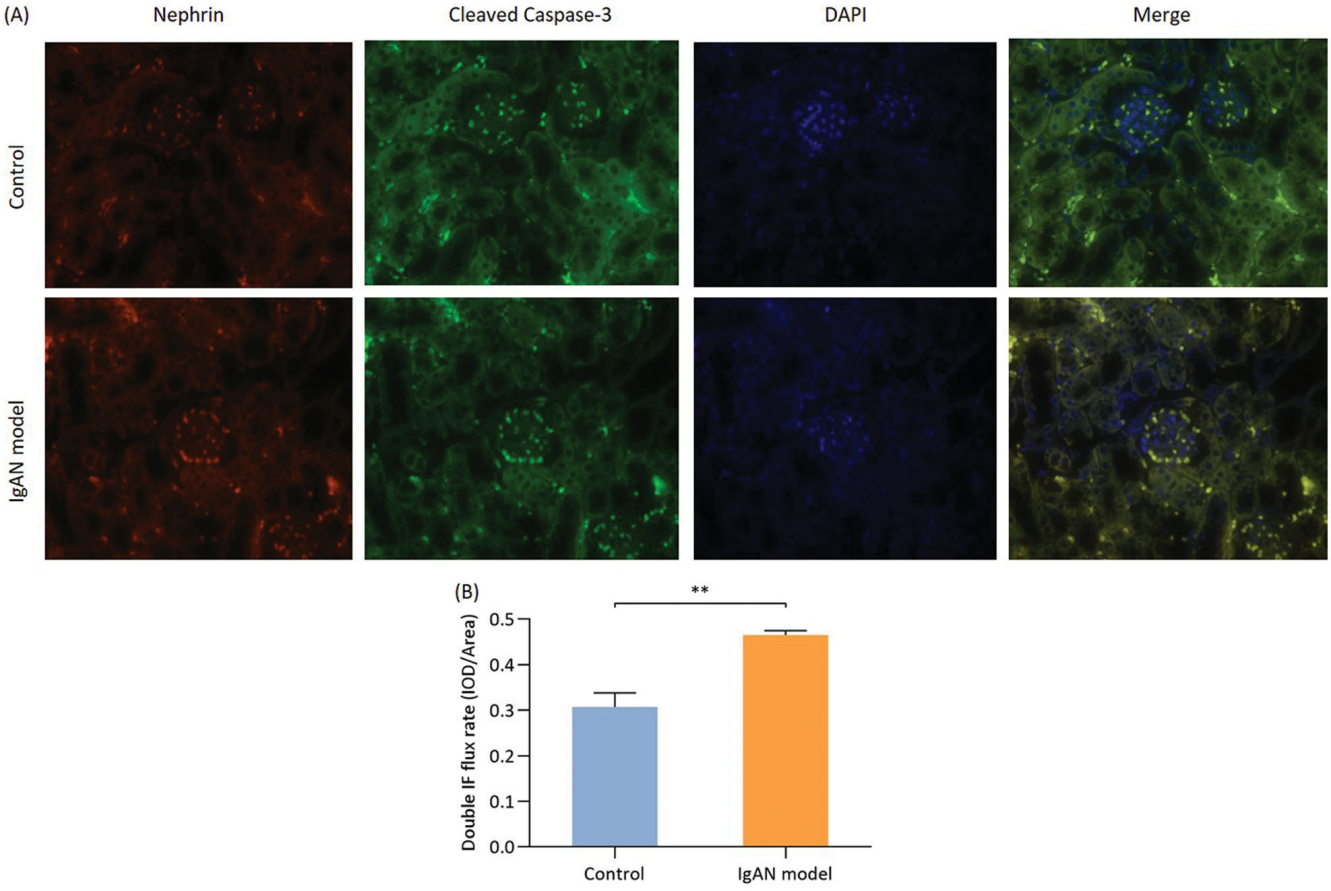
Colocalization of nephrin and cleaved caspase-3 in podocyte apoptosis in IgAN mice. (A) Double immunofluorescence was used to determine the expression of nephrin and cleaved caspase-3 in podocytes (magnification, x400). (B) Double immunofluorescence flux rate. Data are expressed as mean ± SD. *p < 0.05, **p < 0.01 vs. control group. IgAN, IgA nephropathy.

Cleaved-caspase-3 is the activated form of caspase-3. Caspase-3 is a key apoptotic enzyme, that mostly exists in its inactive form. Double immunofluorescence staining of cleaved caspase-3 using FITC fluorescence and of nephrin using ALEXA fluorescence was performed on renal tissue paraffin sections. The results of double fluorescence immunostaining quantified by semi-quantitative analysis revealed that the fluorescence intensity in the IgAN group was significantly higher compared with that of the control group (*p* < 0.01; Figure 5a, b).

### TNF-α induces podocyte apoptosis via the death receptor pathway

**Figure 6.**
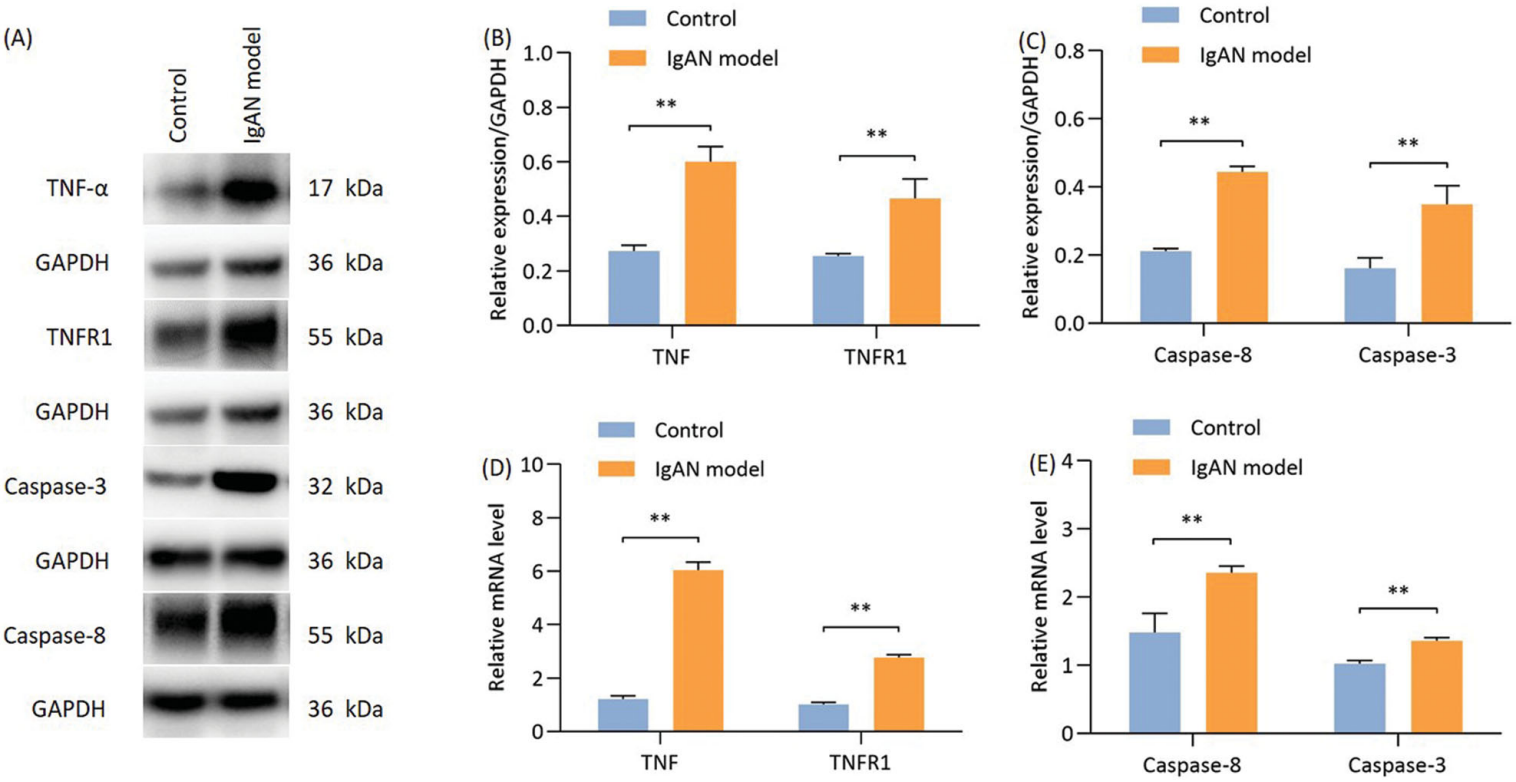
Proteins and mRNA expression of TNF-α, TNFR1, caspase-8 and caspase-3 are significantly reduced in IgAN mouse kidneys. (A) Western blotting results and (B) relative protein expression of TNF-α and TNFR1. (A) Western blotting results and (C) relative protein expression of caspase-8 and caspase-3. (D) Transcription levels of TNF-α and TNFR1 (*n* = 3). (E) Transcription levels of caspase-8 and caspase-3 (*n* = 3). Data are expressed as mean ± SD for groups of 20 mice. *p < 0.05, **p < 0.01 vs. control group. TNFR1, TNF receptor 1; IgAN, IgA nephropathy.

The expression of TNF-α, TNFR1, caspase-8 and caspase-3 in renal tissues was detected by Western blotting. Compared with the control group, the expression of TNF-α and TNFR1 in the renal tissues of the model group was significantly higher, (*p* < 0.05; Figure 6a, b). Furthermore, the expression of caspase-8 and caspase-3 in the kidney tissues of model group mice was significantly higher compared with that in the control group (*p* < 0.01; Figure 6a, c).

The mRNA expression of TNF-α, TNFR1, caspase-8 and caspase-3 in renal tissues was detected using RT-qPCR. The results demonstrated that the expression of TNF-α, TNFR1, caspase-8 and caspase-3 was significantly higher in the model group compared with that in the control group (*p* < 0.01; Figure 6d, e).

## Discussion/conclusion

A variety of IgAN models have been designed and constructed in an attempt to elucidate the complex pathogenesis of IgAN. Rifai *et al* were the first to successfully establish an IgAN animal model in 1979, by using BSA as a vector, with dinitrophenol as the antigen [21]. Next, Isaac *et al* found that negatively charged dextran antigens delivered by intraperitoneal and intravenous injection caused significant IgA, IgM and complement deposition with moderate mesangial cell proliferation [22]. Emancipator [23] and Lai [24] successfully induced an IgAN mouse model using heterologous proteins. Liu and Li [18] successfully constructed IgAN animal models by using SEB intravenous injection into Sprague-Dawley rats in 1989. SEB is a soluble bacterial exotoxin protein. Intravenous injection into the animal can damage the intestinal capillaries, increase intestinal mucosal permeability, increase intestinal exposure to food antigens causing a local and systemic inflammatory response, and increased local IgA secretion. The results demonstrated that the excess levels of serum IgA exceeded the liver transport and clearance capacity, which led to the final deposition of IgA in the renal mesangial area and provided evidence that intestinal mucosal immune abnormalities may result in IgAN.

In our experiment, heterologous protein BSA was administered orally, combined with SEB tail vein injection, which has been investigated in previous studies and is widely used in IgAN experimental research. The results demonstrated that kidney damage was observed at the end of the 6th, week and the 24-h urinary protein level was notably increased. By the end of the 12th week, the levels of SCr and UA were significantly higher in IgAN mice compared with the control group. The immunofluorescence results also revealed IgA immune-complex deposition of varying degrees in the glomerular mesangial area in the IgAN group. Renal pathological examination indicated that the model group mainly exhibited glomerular mesangial cell proliferation and an increase in the mesangial matrix, whereas focal segmental sclerosis and severe glomerular fibrotic changes were also observed in some cases. The association between the glomerular and tubulointerstitial lesions is relatively consistent: When mesangial changes are mild to moderate, there are usually no obvious tubulointerstitial lesions; however, renal tubular epithelial cell vacuolar degeneration and renal interstitial mononuclear cell infiltration may be observed in association with diffuse mesangial proliferation in IgAN mice. The aforementioned results indicated that oral administration of BSA combined with SEB tail vein injection can successfully establish an IgAN mouse model, and the main pathological changes are the IgA immune complex deposition in the mesangial area, with the proliferation of mesangial cells and mesangial matrix, whereas the main symptoms and signs include proteinuria and renal function decline.

Podocytes, the GBM and endothelial cells constitute the glomerular filtration membrane, and the foot processes of neighboring podocytes are interdigitated, forming a complex modified adherens junction, known as the slit diaphragm [6]. Podocyte injury involves apoptosis, necrosis, detachment from the GBM and defective autophagy, ultimately leading to proteinuria [25]. The reduction in the number of podocytes in IgAN is closely associated with disease severity [26]. *In vitro* experiments have revealed that the mesangial cell-derived humoral factor TNF-α can alter glomerular permeability and cause podocyte apoptosis in the event of proteinuria in IgAN [7]. Nephrin is an important podocyte-specific protein and it is involved in signaling pathways regulating actin dynamics and cell survival [27]. Podocin is a structural molecule that interacts with nephrin and they both play an important role in supporting the slit diaphragm [28,29]. However, the pathway and underlying mechanisms through which TNF-α leads to podocyte apoptosis in IgAN have yet to be fully elucidated.

Apoptosis is the most typical form of programmed cell death. The present study demonstrated that podocyte apoptosis may be one of the causes of proteinuria in IgAN mice. Podocytes are an important component of the glomerular filtration barrier that adheres to the GBM [30]. Upon podocyte loss, nonselective proteinuria appears, with ensuing progressive glomerulosclerosis [31]. Podocyte loss was confirmed by the finding that the gene and protein expressions of podocin and nephrin were significantly upregulated, and the number of apoptotic TUNEL positive cells in the glomeruli of IgAN mice was significantly higher compared with that in the control group. Double-staining immunofluorescence revealed co-localization of nephrin and cleaved caspase-3, indicating that podocyte apoptosis was involved in IgAN podocyte injury.

TNF-α has been shown to induce podocyte apoptosis, and blocking TNF-α protects glomerulosclerosis and podocyte apoptosis in hypertensive rodents. However, its exact mechanism is still unclear [32]. The most important finding in the present study is that TNF-α may serve as a therapeutic target for proteinuria in IgAN mice. This was supported by the finding that the gene and protein expressions of TNF-α, TNFR1, caspase-3 and caspase-8 were upregulated in IgAN mice compared with the group. There are two main signal transduction pathways that lead to cell apoptosis: The extrinsic and the intrinsic pathways. In the extrinsic pathway, TNF-α serves as the death ligand, binding with its corresponding death receptor that is embedded in the cell membrane. Death receptors are a class of type I transmembrane proteins that belong to the TNF receptor family [33]. After ligand binding, the membrane death receptor TNFR1 assembles a DISC [34] and initiates the caspase cascade. Once caspase-8 becomes activated, it can then activate the executioner caspase-3. Activation of the caspase cascade causes the degradation of important proteins and the activation of nucleic acid enzymes, ultimately leading to apoptosis [35]. However, many other pathways may play a role. It is reported that up-regulation of ATR1 and PRR expression and the associated activation of NFκB and notch signaling pathway axis are involved in the podocyte apoptosis [15]. Hence, further research is crucially needed to test our hypothesis by giving specific inhibitors or anti-TNF-α therapy to check whether the upregulation of the TNF/TNFR1/caspase-8/caspase-3 death pathway components is the primary cause of podocyte apoptosis.

In conclusion, an IgAN model can be successfully constructed through BSA gavage and SEB tail vein injection. TNF-α may serve an important role in podocyte damage of IgAN mice. It was previously demonstrated that the deposition of IgA immune complexes in human mesangial cells triggers the production of TNF-α and induces podocyte apoptosis [35]. The results of the present study suggested that the release of TNF-α-mediated podocyte injury may be related to the apoptotic death receptor pathway, which is involved in the early pathological changes of the glomerulus in IgAN.
